# Viral and Cell Cycle–Regulated Kinases in Cytomegalovirus-Induced Pseudomitosis and Replication

**DOI:** 10.1371/journal.ppat.0030006

**Published:** 2007-01-05

**Authors:** Laura Hertel, Sunwen Chou, Edward S Mocarski

**Affiliations:** 1 Department of Microbiology and Immunology, University of Western Ontario, London, Ontario, Canada; 2 VA Medical Center, Portland, Oregon, United States of America; 3 Department of Microbiology and Immunology, Emory University School of Medicine, Atlanta, Georgia, United States of America; University of Florida, United States of America

## Abstract

A process of pseudomitosis occurs during human cytomegalovirus infection that appears similar to cellular mitosis but involves the formation of multiple spindle poles, abnormal condensation, and mislocalization of chromosomal DNA. The relationship of this process to viral replication and cell cycle regulation during infection has been poorly understood. Pseudomitosis consistently peaks at late times of infection in all viral strains examined but at overall highest frequencies (30% to 35% of cells) using one common laboratory strain variant (AD169*var*ATCC). Cyclin-dependent kinase 1 (Cdk1) plays a crucial role in pseudomitosis, mirroring its role in conventional mitosis. Dominant negative Cdk1 inhibits and wild-type Cdk1 stimulates this process; however, viral yields remain the same regardless of pseudomitosis levels. Broad inhibition of cell cycle−regulated kinases (Cdk1/Cdk2/Cdk5/Cdk9) with indirubin-3′-monoxime substantially decreases viral yields and synergizes with the viral UL97 kinase inhibitor, maribavir. Thus, Cdk1 is necessary and sufficient to drive pseudomitosis, whereas a combination of viral and cell cycle−regulated kinases is important during viral replication.

## Author Summary

The human herpesvirus cytomegalovirus, which infects most people worldwide, orchestrates a stimulatory effect on cell growth and division, creating an environment that appears similar to many cancer-causing viruses that may be important in viral disease. In previous work, we discovered that viral infection stimulates cells to proceed to an abnormal state mimicking cell division or mitosis but blocks the formation of daughter cells. Here the abnormal state (pseudomitosis) is shown to depend on viral events that drive high levels of the cellular enzyme cyclin-dependent kinase 1 (Cdk1), normally associated with progression through cell division. Although Cdk1 by itself exerts no detectable influence on viral replication levels, host cell cyclin-dependent kinases play an overlapping role with the virus-encoded protein kinase (UL97) in viral replication. Specific inhibitors of these host and viral kinases are used to demonstrate that Cdk1 is necessary and sufficient to drive pseudomitosis; however, viral as well as cell cycle−regulated kinases are important for efficient viral replication.

## Introduction

DNA viruses have yielded many insights into cell cycle control as well as regulation of cellular processes associated with oncogenesis because they encode regulatory proteins that modulate cell cycle progression and cell death. Members of the papillomavirus, polyomavirus, hepadnavirus, and herpesvirus families all cause persistent/latent infections, and, regardless of any contribution to malignancy, all of these viruses encode functions that dysregulate cellular growth, inhibit immune activation, and modulate inflammation [1]. Lifelong infection with human herpesviruses, including herpes simplex virus (HSV)-1, HSV-2, varicella zoster virus, Epstein-Barr virus, cytomegalovirus (CMV), human herpesvirus 6A, 6B, or 7, and Kaposi sarcoma−associated herpesvirus , is associated with dysregulation of the cell cycle as well as with medically important opportunistic disease [2,3]. The mechanisms of cell cycle dysregulation in herpesvirus replication and proliferative disease remain poorly understood even though these processes underlie pathogenesis.

Human CMV has a dramatic stimulatory impact on permissive cells in culture, as typically demonstrated by infecting human fibroblasts (HFs). Stimulation follows a bimodal pattern, with an initial peak due to the impact virus binding and penetration, and a later peak coinciding with viral DNA synthesis, assembly and release [4–7]. This late phase of infection is also associated with dysregulation of host cell cyclin levels and with disruption of cell cycle progression [8–11] and suggests that these processes are linked. Viral DNA replication and cell cycle dysregulation both depend upon expression of viral immediate-early (IE, or α) and delayed-early (DE, or β) gene products, which precede expression of late genes and release of progeny virus [6]. This pattern also implicates early viral gene products in cell cycle stimulation and dysregulation [5,12] in a manner reminiscent of oncogene-encoding, DNA tumor viruses [13]. Although infected cells exhibit a pattern of protein expression consistent with continued cell cycle progression, normal cellular prereplication complexes fail to form [14], cellular DNA synthesis (S phase) fails to proceed [8–11], and chromosome segregation and cytokinesis are blocked [8–11]. These investigations showed that infected HFs become arrested in a pseudo-G1 state associated with dramatic modulation of p53, pRb, cyclins, and cyclin-dependent kinases (Cdks) in ways that may be critical for viral replication efficiency.

The importance of cell cycle–regulated kinases in CMV replication is consistent with a strong antiviral impact of selective Cdk inhibitors such as roscovitine [15]. Roscovitine, as well as other Cdk inhibitors such as olomucine and flavopiridol, inhibits herpesviruses, as well as other DNA viruses and retroviruses, in a pattern that reveals the underlying importance of host cell protein kinases in viral replication [16]. Cdk2 normally complexes with cyclin E or cyclin A in mammalian cells to control G1/S transition. Although cyclin A expression is suppressed [9–11], cyclin E is induced and Cdk2/cyclin E activity is increased [9,17,18] in CMV-infected cells. Most dramatically, the G2/M-associated Cdk1/cyclin B1 complex is induced [8–11] and accumulates at late times of CMV infection [14]. The activity of a wide range of other cell cycle regulators, specific kinases, and cytoskeletal proteins is also altered, and some of these proteins are suspected to function [19–21] during infection [14]. A systematic analysis of cellular gene transcription patterns during infection with a commonly used laboratory strain of CMV (AD169*var*ATCC), provided evidence of extensive cell cycle dysregulation during the late phase of infection, with a peculiar transcriptional enhancement of multiple M-phase regulatory genes [7]. Direct evaluation of infected cultures unveiled the formation of cells with multipolar mitotic spindles and abnormally condensed and misaligned chromosomes, starting by 2 d postinfection. This abnormal process was morphologically distinguishable from conventional mitosis and appeared to represent uncontrolled duplication of centrosomes and aberrant condensation of nonduplicated cellular DNA into mitotic structures [7]. The Cdk1/cyclin B1 complex is a central regulator of entry into M-phase, which ensures the correct segregation of chromosomes into daughter cells at cytokinesis. Regulation of this complex is achieved through the timed synthesis and degradation of cyclin B1 and the tight control of Cdk1 phosphorylation state [22,23]. The elevated Cdk1 and cyclin B1 levels [9,17,18] observed in CMV-infected cells may thus be directly responsible for the induction of pseudomitosis.

The Cdk inhibitor indirubin, the active component of the traditional Chinese anti-inflammatory, antibacterial, and antiviral preparation Danggui Longhui Wan, has also been used to block cell cycle progression [24–26] but has not been evaluated during CMV replication. Indirubin and derivatives such as indirubin-3′-monoxime (IMO), which exhibits improved solubility and cell adsorption qualities, have been used to treat patients suffering from chronic myelocytic or chronic granulocytic leukemias [27]. The antiproliferative properties of this compound depend on high affinity competition for the ATP binding site of Cdk1, Cdk2, and Cdk5 [28], as well as Cdk9 [29]. IMO induces cell cycle arrest at both the G1/S and G2/M borders, consistent with blocking Cdk2/cyclin E and Cdk1/cyclin B1 activities in cultured cells [28] and animal models [30].

Specific inhibition of Cdk1 and Cdk2 activity provided compelling evidence for the function of these kinases at specific stages of the cell cycle. Dominant negative, kinase-inactive Cdk1 arrests cycling cells at the G2/M border, while dominant negative Cdk2 inhibits G1/S progression [31]. The reported inhibition of CMV infection by dominant negative Cdk2 [15] is consistent with a role for this S phase kinase in viral replication.

In addition to relying on cellular Cdks, CMV encodes the herpesvirus-conserved viral protein kinase (VPK) ppUL97, a serine/threonine kinase that plays an important role during CMV replication [32–35]. Mutation of VPK slows viral DNA replication and maturation events in a manner that depends upon cell state and culture conditions. VPK autophosphorylates and potentially acts on viral substrates, such as the CMV DNA polymerase processivity subunit ppUL44, as well as cellular substrates, including the largest subunit of RNA polymerase II and histone H2B [36,37]. VPK phosphorylates in ways that may overlap with cellular Cdks, is required for activation of the antiviral drug ganciclovir [38–40], and is the target of the selective antiviral drug maribavir (MBV, also called 1263W94) [41,42]. Cells infected with wild-type virus in the presence of MBV or with UL97 mutant exhibit defects in viral replication starting at DNA synthesis and extending through late maturation events [32–35].

Whether pseudomitosis is controlled by viral or cellular functions and how this process contributes to viral replication remain unexplored, although observations on infected cells are consistent with some role for elevated Cdk1/cyclin B1 complex activity. Here we show that induction of pseudomitosis is a common property of CMV strains but is not a requisite for replication. Pseudomitosis is specifically dependent on Cdk1 activity, while viral replication requires a broader set of Cdks acting in concert with VPK.

## Results

### Pseudomitosis Is a Common Feature of CMV-Infected Cultures

To determine whether pseudomitosis observed during infection with a previously evaluated CMV strain variant (AD169*var*ATCC) [7] was a universal feature of commonly used CMV strains, we recorded the percentage of pseudomitotic cells induced in HFs infected at a multiplicity of infection (MOI) of 10 with AD169*var*ATCC, AD169*var*DE, Toledo, Towne*var*ATCC, Towne*var*RIT, or TB40/E for 50, 72 and 96 h (Figure 1). Pseudomitotic cells were identified by staining infected cultures for the viral nuclear antigens IE1/IE2 and for the mitotic spindle pole-localizing protein γ-tubulin 1, as described [7]. All strains showed the ability to drive infected cells into pseudomitosis, albeit with different efficiencies and following different temporal patterns. As expected, AD169*var*ATCC induced abnormal numbers of mitotic spindles and misalignment of condensed chromosomal DNA in approximately 35% of infected cells, peaking at 72 h postinfection [7], while all other CMV strains induced pseudomitosis at lower overall levels. Surprisingly, another AD169 strain variant (AD169*var*DE) induced much lower peak levels (7% at 72 h postinfection) compared to AD169*var*ATCC. The only known difference between these strain variants is in the UL36 gene product, which has a point mutation that renders the viral inhibitor of caspase 8 activation nonfunctional [43], suggesting that viral genetic determinants such as this anti-apoptotic function may impact this process. All other strains displayed top levels below 5%, and varied in the timing of maximal pseudomitosis levels, with Toledo and Towne*var*ATCC peaking at 72 h postinfection but Towne*var*RIT and TB40/E showing highest levels at 50 h postinfection. Thus, although induction of pseudomitosis is a common property of CMV strains in culture, AD169*var*ATCC exhibits substantially higher levels than the other strains we examined.

**Figure 1:**
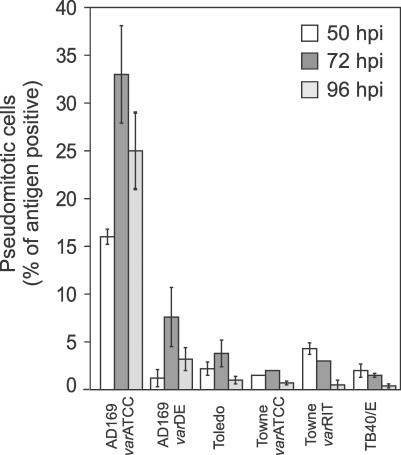
Pseudomitosis Levels Induced by Commonly Used CMV Strains Confluent HFs infected with the indicated CMV strains for 50, 72, and 96 h were immunostained with monoclonal antibodies (mAb) against γ-tubulin 1 and against IE1/IE2. The percentage of IE1/IE2^+^ pseudomitotic cells was determined after counting at least 500 cells for each strain at each time point [7]. Mean percentages and standard deviation from a representative experiment are shown.

### Cdk1 Is Necessary and Sufficient to Promote Pseudomitosis during Infection

The levels of endogenous Cdk1 were induced 30% more in AD169*var*ATCC than in AD169*var*DE infected cells (Figure 2A), consistent with a role for this cyclin-dependent kinase in driving pseudomitosis levels. To determine the extent to which Cdk1 activity alone contributed to the development of pseudomitosis, retrovirus-transduced HFs expressing enhanced green fluorescent protein (GFP), a GFP-tagged, wild-type Cdk1 (Cdk1wt-GFP) or a GFP-tagged, kinase inactive and dominant negative Cdk1 (Cdk1dn-GFP) [31] were infected with AD169*var*ATCC or AD169*var*DE (MOI of 10). Immunoblot analysis showed that both endogenous (black arrowhead) and recombinant Cdk1 (Cdk1wt-GFP or Cdk1dn-GFP; gray arrowhead) were comparably expressed in infected cells at 72 h postinfection (Figure 2A). In all experimental settings, AD169*var*ATCC induced endogenous Cdk1 to higher levels than AD169*var*DE, and, independent of the retrovirus construct, transduction reduced endogenous Cdk1 levels by 20% to 30%. Cdk1dn-GFP or Cdk1wt-GFP was also expressed in cells infected with AD169*var*ATCC or AD169*var*DE, with levels appearing to be substantially higher in AD169*var*DE-infected cells transduced with either construct. Total (endogenous plus transduced) Cdk1 levels were approximately 80% in AD169*var*ATCC- and 160% in AD169*var*DE-infected cells compared to nontransduced cells. No difference was observed in the expression of Cdk1wt-GFP or Cdk1dn-GFP in cells infected with either virus; however, expression was twice as high in AD169*var*DE-infected cells, suggesting a level of variation in total Cdk1 levels that was not explored further.

**Figure 2:**
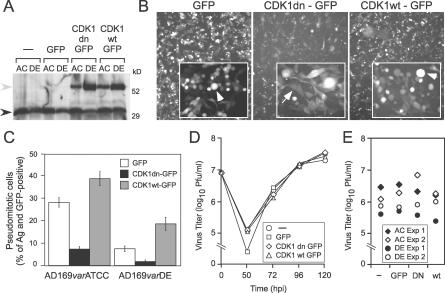
Impact of Cdk1 on Pseudomitosis and Replication (A) Immunoblot analysis of Cdk1 expression in protein extracts from nontransduced (−) and GFP-, Cdk1wt-GFP−, or Cdk1dn-GFP−transduced HFs infected with AD169*var*ATCC (AC) or AD169*var*DE (DE) for 72 h. The blot was incubated with an anti-Cdk1 monoclonal antibody (mAb) as described in Materials and Methods. Expected molecular weights: 34 kDa for Cdk1 and 61 kDa for Cdk1wt or dn-GFP. Black arrowhead, endogenous Cdk1; gray arrowhead, recombinant Cdk1. (B) GFP fluorescence images of HFs transduced with retroviruses expressing GFP, Cdk1wt-GFP, or Cdk1dn-GFP and infected with AD169*var*ATCC for 72 h (original magnification ×50). Higher magnification images (×200) are displayed in the insets. Arrowheads indicate representative pseudomitotic cells; arrows indicate interphase cells. (C) Percentage of IE1/IE2^+^ and GFP^+^ pseudomitotic cells in GFP-, Cdk1wt-GFP−, or Cdk1dn-GFP−transduced HFs infected with AD169*var*ATCC or AD169*var*DE for 72 h. Mean percentages and standard deviation from four independent experiments are shown. (D) Viral yields from nontransduced (−) and GFP-, Cdk1wt-GFP−, or Cdk1dn-GFP−transduced HFs infected with AD169*var*ATCC for 50, 72, 96, and 120 h in one representative experiment. (E) Viral yields from nontransduced (−) and GFP-, Cdk1wt-GFP−, or Cdk1dn-GFP−transduced HFs infected with AD169*var*ATCC or AD169*var*DE for 72 h in two independent experiments.

GFP fluorescence was detectable in 90% to 100% of transduced HFs (Figure 2B) and facilitated differentiation between the round, anucleated pseudomitotic cells and the spindle-shaped, nucleated cells that retained an interphase appearance (Figure 2B, insets). Because of the differences in endogenous and transduced Cdk1 expression levels depending on the strain variant, comparisons were made within a variant, although Cdk1 inhibition or overexpression had similar effects on both AD169*var*ATCC- and AD169*var*DE-infected HFs when IE1/IE2 antigen–positive, GFP-positive, pseudomitotic cells were counted (Figure 2C). High levels of pseudomitotic cells were observed in AD169*var*ATCC-infected HFs following transduction with Cdk1wt-GFP. By contrast, such cells were rare in cultures that had been transduced with Cdk1dn-GFP. When four independent experiments were analyzed together, the peak levels of pseudomitosis in either AD169*var*ATCC- (28 ± 2%) or AD169*var*DE- (7 ± 1%) infected cultures were reduced approximately 4-fold in the presence of Cdk1dn-GFP, indicating that Cdk1 activity was necessary for this process. Conversely, transduction of Cdk1wt-GFP, which increased the total cellular pool of active Cdk1, induced pseudomitosis 1.4-fold with AD169*var*ATCC and 2.5-fold with AD169*var*DE, consistent with expression levels (Figure 2A). Pseudomitosis levels in GFP controls were similar to levels in nontransduced cells, indicating a neutral impact of GFP expression on this process. Thus, Cdk1 appears necessary and sufficient for induction of pseudomitosis in CMV-infected cells, consistent with its role in regulating mitosis in the conventional cell cycle [31].

### Cdk1 and Pseudomitosis Are Dispensable for Viral Replication

To assess the impact of pseudomitosis on viral yields, CMV (AD169*var*ATCC) released into culture supernatants was titered at 50, 72, 96, and 120 h postinfection, comparing nontransduced HFs to HFs transduced with GFP, Cdk1dn-GFP, or Cdk1wt-GFP (Figure 2D). No differences in virus titers were observed at any time in any of these conditions despite the dramatic impact that modulation of Cdk1 levels had on pseudomitosis. In separate experiments, nontransduced HFs or HFs transduced with GFP, Cdk1dn-GFP, or Cdk1wt-GFP and infected with AD169*var*ATCC or AD169*var*DE were found to produce similar amounts of virus at 72 h postinfection (Figure 2E). Thus, the dramatic modulation of pseudomitosis that was observed using these vectors did not contribute to altered virus replication or release. Therefore, pseudomitosis is a reflection of induced Cdk1 activity during infection, but viral replication proceeds with equal efficiency in HFs regardless of Cdk1 activity.

### Viral Replication Depends on the Activity of Cellular Kinases

Given that Cdk1 activity does not influence CMV replication levels, we evaluated the impact of IMO, a Cdk1/Cdk2/Cdk5/Cdk9 inhibitor [28], on CMV replication (MOI = 10) at 24, 48, 72, 96, 120, and 144 h postinfection. IMO has been used to inhibit wild-type and drug-resistant strains of HIV-1 in culture [29], but its antiviral effects against CMV or other herpesviruses had never been tested. Enzyme inhibition studies suggested IMP is a more potent and specific Cdk1 and Cdk2 inhibitor than roscovitine [28,44]. Appearance of progeny virus in culture supernatants was delayed at 5 μM IMO and significantly inhibited at 10 μM IMO regardless of whether AD169*var*ATCC or AD169*var*DE was used (Figure 3A). DMSO (the IMO solvent) at 1% or 2% had no impact on virus levels. The maximum effect of 5 μM IMO (200- to 300-fold reduction) was observed at 72 h postinfection, although viral levels continued to rise. Peak viral yields were obtained at 120 h postinfection, with a delay of 24 h and a 5-fold reduction in titer compared to DMSO-treated cultures. IMO 10 μM inhibition was more dramatic, with a greater than a 10,000-fold reduction at 72 h and greater than 20-fold reduction at 144 h postinfection (Figure 3A). The IMO-specific impact on pseudomitosis could not be assessed because DMSO, the solvent, at either 1% or 2% blocked this process via a mechanism that has not been explored. IMO at 5 or 10 μM (or DMSO solvent alone up to 2%) in medium replaced daily had no effect on uninfected cell viability or total numbers over a 5-d time course (unpublished data). Numbers of virus-infected HFs started to decline between 72 and 96 h postinfection, but this has been considered a normal consequence of infection [45] and the intensity and rate of cell loss were not increased by treatment. Staining with Hoechst 44432 showed that nuclei were intact and presented no evidence of pseudomitosis or other changes in nuclear or chromatin morphology.

**Figure 3:**
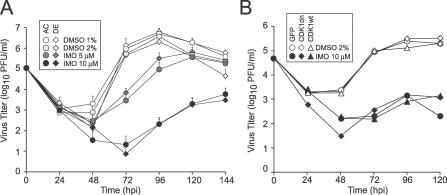
AD169*var*ATCC and AD169*var*DE Yields Depend on the Activity of Cellular Kinases (A) Mean viral yields with standard deviations (three independent experiments) from HFs infected with AD169*var*ATCC or AD169*var*DE in the presence of 1% or 2% DMSO or in the presence of 5 μM or 10 μM IMO for 24, 48, 72, 96, 120, and 144 h. (B) Viral yields from GFP-, Cdk1wt-GFP−, or Cdk1dn-GFP−expressing HFs infected with AD169*var*ATCC in the presence of 2% DMSO or 10 μM IMO for 24, 48, 72, 96, and 120 h from one representative experiment.

Finally, we investigated the effects of combining specific Cdk1 inhibition and IMO treatment on viral replication. Neither IMO (10 μM) nor DMSO (2%) alone altered levels of viral replication in GFP-, Cdk1wt-GFP–, or Cdk1dn-GFP–transduced HFs above those expected of IMO alone (Figure 3B). Expression of transduced proteins was not altered by IMO or DMSO treatment, although pseudomitosis was completely blocked in all of these conditions (unpublished data). Thus, Cdk1 does not independently contribute to viral replication in any of the conditions we used, while a broader set of cellular kinase activities is important for efficient viral cycle progression.

### Contribution of VPK ppUL97 and Cdks to Viral Yields

Treatment of CMV-infected HFs with 10 μM IMO reduced but did not completely block replication levels, a pattern similar to that observed with cells infected with UL97 mutants [32,34,35] or treated with MBV, a potent inhibitor of the UL97-encoded VPK [41,46–48]. Thus, we investigated the combined impact of simultaneously inhibiting cellular Cdks and VPK. To evaluate the contribution of VPK, we titered progeny virus levels released in the supernatant of confluent AD169*var*ATCC-infected HFs that were treated with IMO alone, MBV alone, or combinations of these two inhibitors. IMO was used at the two previously evaluated concentrations (5 μM and 10 μM) in the absence of MBV or together with MBV at subinhibitory (0.025, 0.1, or 0.4 μM) concentrations. Drug-containing medium was replaced daily, and virus titers were determined at 24, 48, 72, 96, and 144 h postinfection (Figure 4). As expected from work describing the activity of this antiviral drug on HFs [49], addition of MBV alone at 0.025 or 0.1 μM did not inhibit viral levels and addition of MBV at 0.4 μM reduced viral levels only 2- to 3-fold. MBV substantially enhanced the antiviral activity of IMO in a dose- and time-dependent manner. The most dramatic effects on viral replication were achieved in the presence of 5 or 10 μM IMO plus 0.4 μM MBV. Specifically, there was a 500-fold enhancement of antiviral activity when 10 μM IMO was used together with 0.4 μM MBV at 72, 96, and 144 h postinfection. These results highlight the dramatic dependence of CMV replication on the activity of both cellular and viral protein kinases. Furthermore, these results demonstrate the potential therapeutic benefit of combining cellular Cdk and VPK inhibitors to treat CMV infections.

**Figure 4:**
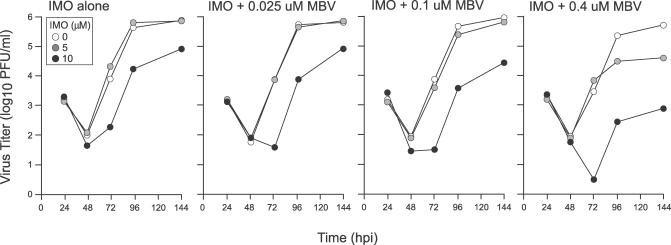
Checkerboard Analysis of Cellular Cdk Inhibitor IMO and VPK Inhibitor MBV on Viral Yields Mean infectivity (with standard deviation of four replicates indicated by error bars) determined by plaque assay on culture supernatants of HFs at 24, 48, 72, 96, and 144 h postinfection with AD169*var*ATCC in the presence of 0, 5, or 10 μM IMO (left) or in combination with different concentrations of MBV (0.025, 0.1, and 0.4 μM) from one experiment.

## Discussion

Whereas the aberrant process leading to pseudomitosis is dependent on Cdk1 levels, replication of CMV appears to require overlapping contributions of Cdks and VPKs. The synergism of a specific inhibitor of CMV VPK and a broad inhibitor of Cdks best illustrated this interdependence. The CMV VPK inhibitor MBV inhibits viral replication in culture, showing a pattern that is reminiscent of VPK mutants [32,34,35]. Herpesvirus-common VPKs are encoded by all mammalian and avian herpesviruses [33]. In HSV-1 and other alphaherpesviruses that are studied in actively growing cell lines, VPKs have been found to be dispensable for replication even though they impact the phosphorylation status of viral proteins and are important for viral replication in the host. In CMV, UL97-encoded VPK provides a critical replication function in quiescent cells, promoting the efficiency of virion maturation at different steps [32–35]. The variable impact on viral replication levels together with the fact that a number of distinct late replication events are sensitive to the absence or inhibition of VPK is consistent with an overlap in VPK and Cdk activities, an overlap that gives the impression that VPK is dispensable in some herpesviruses such as HSV-1 because these viruses are propagated in actively growing cell lines. Similar to the behavior of UL97 mutant virus, the antiviral effect of MBV is influenced by culture conditions and metabolic status of host cells in culture [49]. By exploring the effects of Cdks and VPK inhibition, we have been able to dissect the impact of protein kinases in virus-induced cell cycle dysregulation as well as viral replication. The dependence of CMV-induced pseudomitosis on elevated cellular Cdk1 activity contrasts with the broader, overlapping role of Cdks and VPK in replication. Future work will focus on investigating the behavior of UL97 mutant virus or MBV-treated wild-type virus in the presence of cellular kinase inhibitors to further dissect the role of individual kinases in host cell modulation and viral replication.

Induction of Cdk1 underlies the higher levels of pseudomitosis observed with AD169*var*ATCC and lower levels observed with AD169*var*DE, and both may be modulated with predictable results by transduction of dominant negative Cdk1 or wt Cdk1. The viral function(s) that influence the levels and activity of Cdk1 during infection is unknown, and as such, levels of pseudomitosis may reflect viral regulation of a gene product that drives or inhibits this process. Virus strain and/or cell culture conditions clearly influence pseudomitosis, and one viral function that may play a role, based on characterized differences between strain variants used here, is the cell death suppressor viral inhibitor of caspase 8 activation [43]. The precise roles played by virus-encoded cell death suppressors is starting to emerge [45]; however, interplay between the viral inhibitor of caspase 8 activation and cell cycle dysregulation has not been established. Further understanding will come from the use of additional viral mutants, from the continued study of viral strains with high or low pseudomitotic behavior, and from studies that aim at dissecting the Cdk1-dependent events that are critical to the process. Transduction of wild-type Cdk1 induces levels of pseudomitosis as high as 50%, whereas use of dominant negative Cdk1 reduces levels to below 1% without either treatment altering overall virus yields. These data demonstrate that dysregulation of the cell cycle is associated and compatible with CMV replication but that the virus does not rely on this process for any step in replication. It is likely, though, that this virus might require a range of cellular functions that are made available as a result of driving cells into an M-like state. The efficiency of CMV replication in pseudomitotic cells will become better understood by closer examination of maturation events in these nonnucleated cells. It is worth noting that, although current models of envelopment assume the presence of a nuclear membrane, they are compatible with capsid assembly and virion maturation proceeding through the envelopment step in the absence of a nuclear membrane [33,50].

In CMV-infected HFs, Cdk1/cyclin B induction occurs very early, by 8 h postinfection, and this induction is maintained throughout the time course of infection. There appear to be specific virus-induced mechanisms, including the accumulation of cyclin B1, the inactivation of negative Cdk1 regulators such as Myt1 and Wee1, and the accumulation of Cdk1-activating proteins like Cdc25 [18], that provide specific signals to sustain this complex. Despite this, the Cdk1/cyclin B complex by itself does not support CMV replication. Cdk1/cyclin B activation and phosphorylation of specific substrates have also been reported during infection with other herpesvirus, such as human herpesvirus 7 [51] and Epstein-Barr virus [52]. In HSV-1–infected cells, Cdk1 activity is increased [53] and is required to ensure optimal expression of specific late viral genes [54] but also does not affect viral yields. Except for the reported role of Cdk2 in CMV replication [15] and some previous evaluation of MBV [49], there has been little reported investigation of Cdks acting alone or together with viral kinases in the replication of other herpesviruses.

All herpesviruses encode protein kinases which appear to specifically phosphorylate the same amino acid target sequences recognized by Cdk1 [55]. Although the physiological significance of this molecular mimicry is still unclear, it has been suggested that the viral protein kinases may facilitate capsid transport across the nuclear membrane by phosphorylating key components of the nuclear lamina that are also Cdk1 substrates. Consistent with this hypothesis, a CMV UL97 mutant virus exhibits a severely impaired ability to traverse the nuclear envelope [32] under culture conditions where critical roles in DNA replication and encapsidation are apparently supported by cellular kinases such as Cdks [32,34,35].

Compensatory, overlapping activities of VPK and major Cdks may be important much earlier in infection than VPK mutants indicate. The induction of Cdks at early times postinfection has been associated with accurate processing and accumulation of IE1, IE2, UL36, and UL37x1 transcripts [14] as well as with the hyperphosphorylation of the RNA polymerase II C-terminus [56], functions that are important well before the late phase of replication and that may also be carried out by VPK, a virion tegument protein that is introduced into cells upon entry. Even though pseudomitosis has brought focus to the late phase of replication, the coordinated function of viral and cellular kinases likely contributes to regulation from the initial stages through the late stages of replication. This has suggested broad potential for antiviral strategies targeting Cdks, whether applied to viruses that require dividing cells, such as adenoviruses and papillomaviruses, or to viruses that replicate in nondividing cells, such as HIV-1 and herpesviruses [57]. Cdk inhibitors that range in specificity (roscovitine, flavopiridol, olomucine, and IMO) all inhibit CMV replication [14,15] and would be predicted to synergize with MBV in clinically relevant settings.

Here, we demonstrate the potential for therapeutic targeting of viral and cellular kinases using IMO after first showing it has prominent antiviral effects against CMV. This is particularly important considering that indirubins are already successfully used to treat leukemia [58]. In addition, the synergistic antiviral effects of IMO and MBV shown here suggest that a combination of both drugs could be useful to simultaneously target CMV infection as well as cell proliferation related to cancer. This may prove particularly attractive in certain patient populations where both CMV disease and leukemia are threats, such as leukemic patients recovering from blood and marrow allografts.

## Materials and Methods

### Cells and viruses.

Primary HFs and the 293T cell line (a gift of Dr. Jerry Crabtree, Stanford University) were cultured in Dulbecco's modified Eagle's medium (DMEM; GIBCO/Invitrogen, http://www.invitrogen.com) supplemented with 10% Fetal Clone III (HyClone, http://www.hyclone.com), penicillin G (100 U/ml), streptomycin sulfate (100 μg/ml), L-arginine (0.58 mg/ml), L-glutamine (1.08 mg/ml), and L-asparagine (180 μg/ml), here referred to as medium, as described previously [7,45]. Human CMV strain AD169*var*ATCC (lot 22) was obtained from American Type Culture Collection (ATCC, http://www.atcc.com), AD169*var*DE from Dr. M. Mach (Institute für Virologie, Erlangen, Germany), Towne*var*ATCC from Dr. G. Kemble (MedImmune, Mountain View, California, United States), Towne*var*RIT_3_ from M. Stinski (University of Iowa, Iowa City, Iowa, United States), Toledo from Dr. S. Plotkin (Philadelphia, Pennsylvania, United States), and TB40/E from Dr. C. Sinzger (Institute für Virologie, Tübingen, Germany). Propagation and purification of all strains were as described [59]. Virus titers were determined by plaque assay on HF monolayers in six- or 12-well tissue culture plates.

### HF infection with CMV, IMO, and MBV treatments.

HF monolayers were infected at confluency (day 3 postseeding) with purified virions [7] at an MOI of 10. After virus adsorption for 1 h at 37 °C in 5% CO_2_, the inoculum was removed, and the cells were washed twice with medium prior to the addition of fresh medium. For IMO treatment, medium containing 5 μM or 10 μM IMO or containing 1% or 2% DMSO as control was added to cells after the second wash and replaced every 24 h. IMO was purchased from Sigma (http://www.sigmaaldrich.com) and was dissolved in DMSO (Sigma) at a stock concentration of 1 mM. MBV (GlaxoSmithKline, http://www.gsk.com) was used as described [49] alone or in combination with IMO.

### Retrovirus production and cell transduction.

Cdk1wt and Cdk1dn were amplified by PCR from plasmids pCMV-Cdc2-WT or pCMV-Cdc2-D146N (a gift from Dr. S. Van den Heuvel, MGH Cancer Center, Harvard Medical School, Charlestown, Massachusetts, United States) using primers specific for the 5′ and 3′ nucleotide sequence of Cdk1 and containing the BamH1 restriction endonuclease recognition site. Primer sequences were as follows: 5′-TCggatccATGGAAGATTATA-3′ and 5′-ggatccGACTTCTTAATCTGATTG-3′ (BamH1 site in lowercase). PCR bands were inserted in the pCR 2.1-TOPO vector of the TOPO TA Cloning kit (Invitrogen, http://www.invitrogen.com) following the manufacturer's instructions. pCR 2.1-Cdk1wt and pCR 2.1-Cdk1dn were subsequently digested with BamH1, and DNA fragments corresponding to Cdk1wt and Cdk1dn were ligated to the BamH1-digested plasmid LNCX-GFP [45], generating the constructs LNCX-Cdk1wt-GFP and LNCX-Cdk1dn-GFP with each Cdk1 inserted in frame with the nucleotide sequence coding for the N-terminus of GFP. The correct sequence of both constructs was confirmed by nucleotide sequencing (Stanford Protein and Nucleic Acid Facility). To generate retroviral particles, 293T cells were co-transfected with LNCX-GFP or LNCX-Cdk1wt-GFP or LNCX-Cdk1dn-GFP together with plasmids JK3, LTRVSVG, and CMVtat. Cell supernatants were collected at 48 h posttransfection, and retroviral particles were purified and concentrated by ultracentrifugation at 24,000 rpm for 1 h at 4 °C using a SW28 rotor in a Beckman ultracentrifuge (Beckman Coulter, http://www.beckmancoulter.com). Pelleted viral particles were suspended in medium, aliquoted, and stored at −80 °C. For retrovirus transduction, confluent HFs were split at a 1:2 ratio on day 0 and again at a 1:4 ratio on day 1. At 24 h after the second seeding, cells were exposed to the first inoculum of retroviral particles, in the presence of 5 μg/ml Polybrene (Sigma). Then, 5 h later, cells were washed with fresh medium and a second inoculum was applied for another 5 h. At 24 h after removal of the second inoculum, cells were tested for GFP, Cdk1wt-GFP, or Cdk1dn-GFP expression before infection with CMV as described above.

### Immunofluorescence analyses and imaging.

γ-Tubulin 1 and IE1/IE2 double staining was carried out as described [7]. Phase-contrast micrographs of GFP^+^ live cells were obtained with a Nikon Eclipse TE300/200 inverted microscope. For the visualization of GFP and IE1/IE2 double-positive pseudomitotic cells, samples were fixed with 1% paraformaldehyde in PBS for 15 min at room temperature, permeabilized, and blocked as described [7] before staining with an unconjugated monoclonal antibody against IE1/IE2 (1:800, MAb810; Chemicon, http://www.chemicon.com) followed by a Texas red–conjugated goat anti-mouse antibody (1:100; Vector Laboratories, http://www.vectorlabs.com). Cellular DNA was highlighted with Hoechst 33342 (Molecular Probes, http://www.invitrogen.com). Coverslips were mounted and analyzed as described [7].

### Immunoblot analysis.

Cells were suspended in lysis buffer containing 50 mM Tris-HCl (pH 6.8), 2% SDS, 10% glycerol, 5% β-mercaptoethanol, 25 mM sodium fluoride, 1 mM sodium orthovanadate, and 1 mM phenylmethylsulfonyl fluoride. Samples were boiled for 5 min, separated by SDS–polyacrylamide gel electrophoresis through a 7.5% gel, transferred to a polyvinylidene difluoride membrane (Millipore, http://www.millipore.com), and incubated with an anti-Cdk1 monoclonal antibody (1:200; Santa Cruz Biotechnologies, http://www.scbt.com) for 1 h at room temperature before incubation with a horseradish peroxidase–conjugated goat anti-mouse secondary antibody for another hour. Signal was developed by enhanced chemiluminescence (Pierce Biotechnology, http://www.piercenet.com).

### Checkerboard titration of MBV-IMO drug combinations.

HFs (passage 11) were seeded onto 24-well culture plates and maintained in medium until confluent at a density of 1.9 × 10^5^ cells per well. Wells were inoculated with 2 × 10^6^ PFU (in 0.5 ml) of AD169*var*ATCC (MOI = 10). Virus was adsorbed for 1 h at 37 °C in 5% CO_2_, and the inoculum was removed and replaced with 0.5 ml of fresh medium containing combinations of MBV at 0, 0.025, 0.1, and 0.4 μM and IMO at 0, 5, and 10 μM in a checkerboard layout. At 24, 48, 72, 96, and 144 h after inoculation, the culture supernatant was harvested from each well, mixed with 0.5% sterile milk, and frozen at −80 °C until virus titers were determined by plaque assay. Fresh medium containing freshly made drug combinations was replaced in each culture wells until the next collection time point. The checkerboard experiment was performed in triplicate.

## Supporting Information

### Accession Numbers

The UniProtKB/Swiss-Prot (http://www.pir.uniprot.org) accession numbers for the proteins discussed in this paper are Cdk1 (entry name: CDC2_HUMAN, primary accession number: P06493) and AD169 UL97 (entry name: GCVK_HCMVA, primary accession number: P16788).

## Abbreviations

Cdk: cyclin-dependent kinase
CMV: cytomegalovirus
DE: delayed-early
GFP: green fluorescent protein
HF: human fibroblast
HSV: herpes simplex virus
IE: immediate-early
IMO: indirubin-3′-monoxime
MBV: maribavir
MOI: multiplicity of infection
VPK: viral protein kinase
